# Early impact of the DREAMS partnership on young women’s knowledge of their HIV status: causal analysis of population-based surveys in Kenya and South Africa

**DOI:** 10.1136/jech-2020-216042

**Published:** 2021-09-13

**Authors:** Isolde Birdthistle, Daniel J Carter, Nondumiso T Mthiyane, Benedict O Orindi, Sheru Muuo, Natsayi Chimbindi, Abdhalah Ziraba, Maryam Shahmanesh, Kathy Baisley, Sian Floyd

**Affiliations:** 1 Epidemiology & Population Health, London School of Hygiene & Tropical Medicine, London, UK; 2 Public Health & Policy, London School of Hygiene & Tropical Medicine, London, UK; 3 Departments of Population and Social Sciences, Africa Health Research Institute, Durban, Kwa-Zulu Natal, South Africa; 4 Department of Epidemiology & Demography, Kenya Medical Research Institute, Kilifi, Kenya; 5 Health and Systems for Health, African Population and Health Research Center, Nairobi, Kenya; 6 Institute for Global Health, University College London, London, UK

**Keywords:** adolescent, epidemiology, HIV, inequalities, sexual health

## Abstract

**Background:**

Knowledge of one’s HIV status is the gateway to treatment and prevention, but remains low among young people. We investigated the early impact (2016–2017) of Determined, Resilient, Empowered, AIDS-free, Mentored and Safe (DREAMS), a multisectoral HIV prevention package, on knowledge of HIV status among adolescent girls and young women (AGYW).

**Methods:**

In 2017, randomly selected AGYW were enrolled into surveys, N=1081 aged 15–22 years in Nairobi slum settlements, and N=2174 aged 13–22 years in rural KwaZulu-Natal. We estimated the causal effect of being a DREAMS beneficiary on knowledge of HIV status (those who self-reported as HIV-positive or tested HIV-negative in the past year), accounting for an AGYW’s propensity to be a DREAMS beneficiary.

**Results:**

In Nairobi, knowledge of HIV status was higher among DREAMS beneficiaries compared with non-beneficiaries (92% vs 69%, adjusted OR=8.7; 95% CI 5.8 to 12.9), with DREAMS predicted to increase the outcome by 28%, from 65% if none were a DREAMS beneficiary to 93% if all were beneficiaries. The increase attributable to DREAMS was larger among younger participants: 32% and 23% among those aged 15–17 and 18–22 years, respectively. In KwaZulu-Natal, knowledge of status was higher among DREAMS beneficiaries aged 13–17 years (37% vs 26% among non-beneficiaries), with a 9% difference due to DREAMS (95% CI 4.8% to 14.4%), and no evidence of effect among 18–22 years (−2.8%; 95% CI −11.1% to 5.7%).

**Conclusion:**

DREAMS substantially increased knowledge of HIV status among AGYW in Nairobi, and among younger but not older AGYW in KwaZulu-Natal. Adolescent girls can be reached early (before age 18) with community-based HIV testing programmes in diverse high-prevalence settings, with a large impact on the proportion who know their HIV status.

## Introduction

Persistently high rates of HIV infection among young people, particularly young women, have led to large investments in targeted HIV prevention in eastern and southern Africa. This includes the ‘Determined, Resilient, Empowered, AIDS-free, Mentored and Safe (DREAMS) Partnership’ launched in 2015 by the United States’ President’s Emergency Plan for AIDS Relief (PEPFAR) and private sector partners to promote ‘DREAMS’ lives among adolescent girls and young women (AGYW). Through a ‘core package’ of 12 evidence-based interventions, DREAMS promotes a multisectoral approach to reduce HIV incidence and the biological, behavioural, social and economic drivers of young women’s heightened risk.^1^ DREAMS is delivered in districts across 15 countries, with districts selected on the basis of HIV prevalence and incidence estimates.

Enabling young people to know their HIV status is an important precursor to halting new infections. Knowledge of a positive status is a critical first step in linking to life-saving treatment and achieving viral suppression, to improve the health of people living with HIV/AIDS (PLWHA) and to reduce onward transmission. Similarly, confirmation of a negative HIV status can link individuals to information and prevention services, including highly efficacious pre-exposure prophylaxis, to remain uninfected.^2^

Expanded coverage of HIV testing services (HTS) has accelerated progress in global testing and treatment goals, including the ambition for 90% of PLWHA to know they are positive by 2020 (ie, the ‘first 90’, in the 90-90-90 ‘fast track’ targets of the Joint United Nations Programme on HIV and AIDS).^3^ During 2015–2017, the overall proportion of HIV-positive individuals who knew their status rose from ~66% to ~75% globally.^2^ Nevertheless, testing remains the largest gap in the HIV treatment cascade (between current levels and the target of 90%), and the gap is largest among young people, the rapidly growing demographic group on whom epidemic control will depend.^4 5^ In sub-Saharan Africa, less than half of 15–24 years were aware of their HIV status in 2017.^2^

The testing gap among young people suggests they are not sufficiently reached by traditional testing modes. For example, the most common mode, health facility-based testing, is not always available or acceptable to adolescents. A recent systematic review of uptake of HTS among children and adolescents (ages 5–19) concluded that ‘approaches evaluated to date have not been tailored to needs of this age group. Rather, they replicate strategies for adults and do not consider the specific barriers that adolescents face.’^6^ The review noted that many young women first learn their status when they become pregnant, through routine testing in antenatal care. To achieve UNAIDS’ aspiration for universal access to HIV testing, approaches must be taken to enhance supply, demand and uptake among adolescents and young adults.

As part of an ongoing independent impact evaluation of DREAMS,^7^ we sought evidence of an early impact on knowledge of HIV status. HIV testing and counseling services are included in the DREAMS core package, to be linked (‘layered’) appropriately with biomedical, social and educational interventions.^1^ Here, we investigate the effect of participation in DREAMS on knowledge of HIV status among AGYW in two settings, urban Kenya and rural South Africa, after 1 year of DREAMS implementation. We also sought to elucidate the mechanisms by which DREAMS promotes HIV testing in each setting, for example, through different DREAMS delivery models and the influence of contextual factors on implementation.

## Methods

### Study design and settings

In demographic surveillance sites in Nairobi, Kenya and KwaZulu-Natal, South Africa, we selected random samples of AGYW, stratified by age, for cohort studies for the impact evaluation of DREAMS over 3 years of follow-up.^8 9^ For this analysis, we used 2017 enrolment data from the urban informal settlements of Korogocho and Viwandani in Nairobi, (n=1081 aged 15–22 years), and from uMkhanyakude district in KwaZulu-Natal, (n=2174 aged 13–22). Sample size was calculated to ensure statistical power to compare DREAMS beneficiaries with non-beneficiaries across multiple outcomes and a range of assumptions about DREAMS uptake, within two age groups: 13–17 and 18–22 years in uMkhanyakude; and 15–17 and 18–22 years in Nairobi (where 13–14 years were included in a separate ‘early adolescent’ cohort aged 10–14 years, building on the Global Early Adolescent Study pilot in this setting, and not included in this analysis).^7^ Questionnaires were administered in early/mid-2017, approximately 1 year after DREAMS implementation began. Data were collected electronically on a tablet by an interviewer, with the exception of sexual relationship questions which were self-filled in uMkhanyakude for privacy. Further details of the study settings, including sample size calculations, sampling strategies and plans for cohort follow-up, are described in the study protocol.^7^

In both settings, DREAMS interventions were first introduced in 2016, following efforts to identify and recruit vulnerable AGYW (through a door-to-door roster of AGYW supplemented with referrals by community-based organisations in Nairobi, and through geographical mapping to identify high-incidence districts in South Africa, in which all AGYW were considered eligible for DREAMS). Early implementation of interventions was staggered, with services that had a pre-existing infrastructure being the first to roll-out, for example, through expanded or targeted services to include adolescent girls (aged 15–19 years) and young women (20–24 years). More detailed descriptions of DREAMS early implementation, including differences by context, have been summarised previously.^10^

### Measurement of intervention and outcome

We used receipt of an invitation to DREAMS (self-reported, yes or no) as the measure of being a DREAMS beneficiary, regardless of whether or how many services were accessed, analogous to an ‘intention-to-treat’ comparison for a randomised controlled trial study design.^11 12^ An AGYW was defined as knowing her HIV status if she self-reported as HIV positive or she reported having had an HIV test and received the result within the last 12 months (regardless of whether the HIV test was perceived to be affiliated with the DREAMS programme or not).

### Causal effect of being a dreams beneficiary on knowledge of HIV status

The research question is ‘Did DREAMS cause an increase in the proportion of AGYW who know their HIV status, in one or both of the study settings?’ Phrased with respect to a counterfactual outcome, we ask ‘What proportion of AGYW would know their HIV status under two hypothetical scenarios: one in which no individuals were a DREAMS beneficiary, the other in which all individuals were a DREAMS beneficiary.’

### Statistical analysis

#### Sources and measurement of confounding

**Figure 1:**
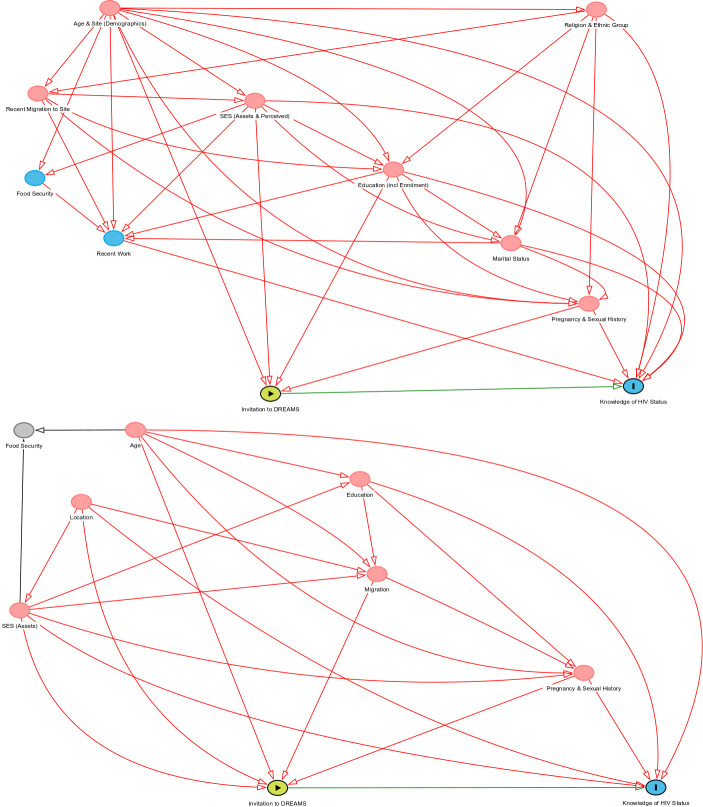
Directed acyclic graphs to identify causal pathways and confounding factors in Nairobi (A) and uMkhanyakude (B). DREAMS, Determined, Resilient, Empowered, AIDS-free, Mentored and Safe; SES, socioeconomic status.

We constructed setting-specific directed acyclic graphs (DAGs) to represent the underlying causal structure of the relationship between being a DREAMS beneficiary, knowledge of HIV status, and other individual and household-level characteristics. In the DAGs in figure 1, each node represents a construct that is measured by ≥1 characteristic recorded from responses to the interview questionnaires. Guided by the work of Greenland *et al*,^13^ each DAG was interrogated to identify the minimal set of constructs that needed to be adjusted for to control for confounding.

We calculated the observed proportion of AGYW who knew their HIV status, by age, site and self-reported DREAMS invitation, and estimated associations between DREAMS invitation and knowledge of HIV status and DREAMS invitation, using logistic regression to adjust for a priori confounders (age and site) and for additional confounding variables informed by the DAGs.

#### Causal effect estimation

To obtain a causal estimate corresponding to the research question of interest (articulated above), we used propensity score logistic regression adjustment. In our analysis, the propensity score is the ‘propensity to be a DREAMS beneficiary’, and is defined as the probability of being a DREAMS beneficiary given a set of individual and household characteristics.^14 15^ The propensity scores were estimated through logistic regression, with the outcome being a DREAMS beneficiary (invited to participate in DREAMS: yes or no), and explanatory variables identified as confounding variables from the DAG or as determinants of knowledge of HIV status. In both study sites, we used a logistic regression model to estimate the propensity score that assumed that the effects of all explanatory variables combined multiplicatively, that is, that there was no effect modification on the log(odds) scale.

Propensity score logistic regression adjustment was undertaken by fitting a logistic regression model for the outcome of knowledge of HIV status, separately for AGYW who were and those who were not DREAMS beneficiaries, with adjustment for the propensity score and age group. These two logistic regression models were then used to predict the probability of the outcome of ‘knowledge of HIV status=yes’ for all AGYW, first under the scenario that they were not a DREAMS beneficiary, and second under the scenario that they were a DREAMS beneficiary. This generated two predicted probabilities for each individual. For each scenario, the predicted probabilities were averaged across all AGYW and also separately for older and younger AGYW. These were then used to calculate the marginal percent difference (the difference in the average predicted probability between the scenario in which all AGYW were DREAMS beneficiaries and the scenario in which none were DREAMS beneficiaries), as well as the corresponding marginal prevalence (risk) ratios and ORs.

CIs were generated by using a bootstrap procedure, repeating the estimation procedure described above in 1000 samples that were drawn with replacement from the complete dataset and calculating 95% CIs from the resulting bootstrap distribution using the 2.5% and 97.5% percentiles.

Given the potential for DREAMS to have a different impact by age group—since DREAMS targeted adolescent girls (15–19 years) and young women (20–24 years) differently—the propensity score adjustment approach was applied within age groups, as well as overall, with a formal test for effect modification by age group.

The assumptions under which our analyses provide a valid causal estimate are defined and examined in online supplemental file 1. To examine the robustness of the causal effect estimates obtained with different assumptions, in addition to propensity score logistic regression adjustment we also use three alternative approaches, namely: propensity score stratification, propensity score weighting and ‘conventional’ multivariable logistic regression (see online supplemental file 1) for the methods used).

10.1136/jech-2020-216042.supp1 Supplementary data

Descriptive analyses and regression analyses for the Nairobi setting were conducted in R V.3.4.1 (R Core Team, 2017). All analyses for the South African setting were conducted in Stata/SE 15.1 software (StataCorp, TX).

### HIV testing modalities and attitudes

For insight into the delivery of HIV testing in DREAMS settings, we summarised respondents’ self-reported experiences and modalities of HIV testing over the previous 12 months, comparing those invited and not invited into DREAMS, separately for two age groups. We also reviewed process evaluation data—drawn from structured observations, key informant interviews with DREAMS implementers and small group discussions with community and family groups and young people, and in-depth interviews with beneficiaries in the first year of programme implementation (data described previously)^10^—to summarise the approach to delivering HIV testing through DREAMS in each context.

## Results

In South Africa, 1148 AGYW aged 13–17 and 1036 aged 18–22 were recruited into closed cohorts (response rate of 85% for 13–22 year olds, of 2555 eligible). In Nairobi, 547 AGYW aged 15–17 and 534 aged 18–22 were enrolled (response rate of 61% of n=1770 eligible 15–22 years).

Almost half (n=536; 49.6%) of the 1081 participants in Nairobi had been invited to participate in DREAMS in the past year: 59% of 15–17 years and 40% of 18–22 yeas (table 1A). This compares to 29% of all AGYW (n=636/2174) in uMkhanyakude, where younger adolescent girls were also more likely than young women to be recruited into DREAMS (40% vs 17%, 13–17 and 18–22 years, respectively) (online supplemental tables S1a-b).

10.1136/jech-2020-216042.supp2 Supplementary data

**Table 1:** Characteristics of DREAMS beneficiaries and non-beneficiaries according to factors identified in the DAG, in Nairobi (A) and uMkhanyakude (B)

(A) Nairobi	Total		DREAMS beneficiaries		Non-beneficiaries	
	N	% (col)	N	% (col)	N	% (col)
Age group						
15–17 years	547	50.6	322	60.07	225	41.28
18–22 years	534	49.4	214	39.93	320	58.72
Age						
15 years	156	14.43	94	17.54	62	11.38
16 years	203	18.78	126	23.51	77	14.13
17 years	188	17.39	102	19.03	86	15.78
18 years	138	12.77	76	14.18	62	11.38
19 years	95	8.79	37	6.9	58	10.64
20 years	102	9.44	41	7.65	61	11.19
21 years	94	8.7	31	5.78	63	11.56
22 years	105	9.71	29	5.41	76	13.94
Location						
Korogocho	617	57.08	314	58.58	303	55.6
Viwandani	464	42.92	222	41.42	242	44.4
Current school status						
Currently in school	626	57.91	371	69.22	255	46.79
Not in school	455	42.09	165	30.78	290	53.21
Highest education						
Primary incomplete	125	11.56	55	10.26	70	12.84
Primary grade 8	217	20.07	92	17.16	125	22.94
Secondary form 1	154	14.25	96	17.91	58	10.64
Secondary form 2	187	17.3	110	20.52	77	14.13
Secondary form 3	150	13.88	86	16.04	64	11.74
Secondary form 4	198	18.32	74	13.81	124	22.75
Tertiary	50	4.63	23	4.29	27	4.95
Household assets						
Low asset score	361	33.4	190	35.45	171	31.38
Middle asset score	360	33.3	172	32.09	188	34.5
High asset score	360	33.3	174	32.46	186	34.13
Poverty perception						
Very poor	139	12.86	77	14.37	62	11.38
Moderately poor	858	79.37	424	79.1	434	79.63
Not poor	84	7.77	35	6.53	49	8.99
Credit access						
No access to credit	550	50.88	265	49.44	285	52.29
Access to credit	483	44.68	250	46.64	233	42.75
Don't know	48	4.44	21	3.92	27	4.95
Ever had sex						
Ever had sex	437	40.43	157	29.29	280	51.38
Never had sex/undisclosed	644	59.57	377	70.34	267	48.99
Ever pregnant						
Ever pregnant	299	27.66	101	18.84	198	36.33
Never had sex	644	59.57	379	70.71	265	48.62
Ever had sex, never pregnant	138	12.77	56	10.45	82	15.05

(B) uMkhanyakude	Total		DREAMS beneficiaries		Non-beneficiaries	
	N	% (col)	N	% (col)	N	% (col)
Age category						
13–17 years	1143	52.6	460	72.33	683	44.44
18–22 years	1030	47.4	176	27.67	854	55.56
Age						
13 years	236	10.86	88	13.84	148	9.63
14 years	222	10.22	76	11.95	146	9.5
15 years	232	10.68	102	16.04	130	8.46
16 years	240	11.04	103	16.19	137	8.91
17 years	213	9.8	91	14.31	122	7.94
18 years	243	11.18	63	9.91	180	11.71
19 years	228	10.49	47	7.39	181	11.78
20 years	242	11.14	36	5.66	206	13.4
21 years	174	8.01	21	3.3	153	9.95
22 years	143	6.58	9	1.42	134	8.72
Location						
Rural	1383	64.21	457	72.66	926	60.72
Periurban/urban	771	35.79	172	27.34	599	39.28
Socioeconomic status						
Low	720	34.88	248	40.52	472	32.51
Middle	746	36.14	209	34.15	537	36.98
High	598	28.97	155	25.33	443	30.51
Currently in school						
No	538	24.76	66	10.38	472	30.71
Yes	1635	75.24	570	89.62	1065	69.29
Level of education						
Primary	206	9.49	75	11.79	131	8.53
Secondary	1628	74.99	520	81.76	1108	72.18
Completed matric	337	15.52	41	6.45	296	19.28
Ever migrated						
No	1771	81.5	564	88.68	1207	78.53
Yes	402	18.5	72	11.32	330	21.47
Food insecurity						
No	1495	68.8	495	77.83	1000	65.06
Yes	678	31.2	141	22.17	537	34.94
Ever been/currently pregnant						
No	1570	73.95	539	85.83	1031	68.96
Yes	553	26.05	89	14.17	464	31.04
Sexually active						
Ever pregnant	553	26.18	89	14.26	464	31.18
Never pregnantandsexually active	248	11.74	58	9.29	190	12.77
Never pregnantandnot sexually active	39	1.85	10	1.6	29	1.95
Never had sex	1272	60.23	467	74.84	805	54.1

DAG, directed acyclic graphs; DREAMS, Determined, Resilient, Empowered, AIDS-free, Mentored and Safe.

Table 1A, B presents the proportion of AGYW who knew their HIV status, and differences in this outcome by DREAMS invitation and individual and household characteristics that comprised the minimal set of explanatory variables that should be adjusted for to control for confounding. This included age, site (slum area), education, indicators of socioeconomic status and position, and sexual and pregnancy history in Nairobi (table 1A). In uMkhanyakude, the confounding set was the same, with location distinguishing urban or rural in this setting (table 1B).

Overall, young women’s knowledge of HIV status was higher in Nairobi, Kenya, where 81% (n=872) of 1081 participants were tested in the past year and/or knew they were HIV-positive, compared with 45% (n=977) of 2173 respondents surveyed in uMkhanyakude, South Africa. Knowledge of status increased with age in both settings: rising from 73% to 91% between the ages of 15 and 22 years in Nairobi, and from 14% to 65% between ages 13 and 22 years in uMkhanyakude. In Nairobi, the strongest association between an individual or household characteristic and knowledge of HIV status was with invitation to DREAMS (age-site-adjusted OR=7.3, 95% CI 4.99 to 10.75), followed by sexual/reproductive history, age, perception of household poverty, area of residence and education (table 1A). In uMkhanyakude, the association between invitation to DREAMS and knowledge of HIV status was much weaker than in Nairobi (age-location-adjusted OR=1.27, 95% CI 1.03 to 1.57), with strong evidence of associations with sexual/reproductive history and age, and weak evidence of an association with food security (table 1B).

In age-stratified analysis, the association between the outcome and history of pregnancy was greater among the younger than the older girls, with 81% of 13–17 years knowing their status if they had been pregnant compared with only 27% among those who had never been pregnant (OR=7.48, 95% CI 3.84 to 14.57; online supplemental table S1a). Urban or rural location was associated with knowledge of status among the younger girls in KwaZulu-Natal, with 13–17 years in urban/periurban areas less likely than those in rural areas to know their status (OR=0.78, 95% CI 0.58 to 1.03; online supplemental table S1a), but there was no evidence of such association among the 18–22 years (online supplemental table S1b).

### Causal effect of DREAMS invitation on knowledge of HIV status

**Figure 2:**
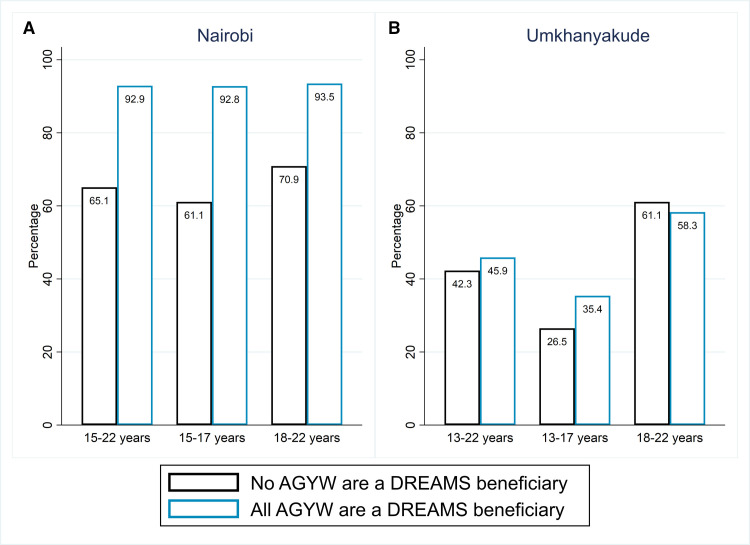
Proportion of AGYW who know their HIV status if none versus all benefit from DREAMS, by age and setting. AGYW, adolescent girls and young women; DREAMS, Determined, Resilient, Empowered, AIDS-free, Mentored and Safe.

In Nairobi, knowledge of HIV status was higher among AGYW who were DREAMS beneficiaries compared with AGYW who were not DREAMS beneficiaries (92% vs 69%; adjusted OR=8.7, 95% CI 5.8 to 12.9; table 2). In propensity score regression adjusted analysis, DREAMS was estimated to increase knowledge of HIV status by 28%, from 65% if no AGYW were a DREAMS beneficiary to 93% if all were a DREAMS beneficiary (95% CI 22.8 to 32.6; table 3, figure 2). This represents the per cent difference attributable to DREAMS (the ‘marginal causal percent difference’), after controlling for confounding variables using regression adjustment for the propensity score. The effect was larger among younger than older participants: 93% vs 61% among beneficiaries vs non-beneficiaries aged 15–17 years (32% difference attributable to DREAMS; 95% CI 24.9 to 39.1) and 94% vs 71% (23% difference due to DREAMS, 95% CI 15.0 to 29.7) among those aged 18–22 years. Alternative measures of effect, including marginal prevalence and ORs, also provided strong evidence that DREAMS had an effect on knowledge of HIV status (table 3).

**Table 2:** Association of DREAMS with knowledge of HIV status using conventional logistic regression: unadjusted risk (%) difference and unadjusted and adjusted ORs, overall and stratified by age

	Not a DREAMS beneficiary (N/%)	DREAMS beneficiary (N/DREAMS beneficiary (N/%)	% Non-beneficiaries know status	% Beneficiaries know status	% Difference (unadjusted)	Unadjusted OR (95% CI)	Ageandlocation adjusted OR (95% CI)	Fully adjusted OR* (95% CI)	P value (LR-test)
Nairobi†									
Overall‡	545(50.4)	536(49.6)	69.36	92.16	22.80	5.20 (3.61 to 7.48)	7.32 (4.99 to 10.75)	8.69 (5.84 to 12.93)	<0.001
15–17 years§	225(41.1)	322(58.9)	54.67	90.37	35.70	7.78 (4.94 to 12.26)	7.90 (4.99 to 12.51)	9.45 (5.82 to 15.36)	<0.001
18–22 years§	320(59.9)	214(40.1)	79.69	94.86	15.17	5.20 (3.61 to 7.48)	7.32 (4.99 to 10.75)	8.69 (5.84 to 12.93)	<0.001
uMkhanyakude†									
Overall¶	1537(70.4)	636(29.3)	46.3	41.8	−4.5	0.84 (0.69 to 1.01)	1.27 (1.03 to 1.57)	1.40 (1.12 to 1.75)	0.003
13–17 years**	683(59.8)	460(40.2)	26.2	36.7	10.5	1.64 (1.27 to 2.11)	1.50 (1.15 to 1.97)	1.61 (1.22 to 2.15)	0.001
18–22 years**	854(82.9)	176(17.1)	62.3	55.1	−7.2	0.74 (0.54 to 1.03)	0.84 (0.60 to 1.18)	0.94 (0.65 to 1.37)	0.749

*Adjusted for minimal set of confounders identified in the DAGs (see final column of online supplemental tables S1a, b and S2a, b) for details).

†Statistical test for interaction provided evidence for interaction by age group in uMkhanyakude (p<0.001) and not in Nairobi (p=0.886).

‡See online supplemental table S1a for more details.

§See online supplemental table S2a for more details.

¶See online supplemental table S1b for more details.

**See online supplemental table S2b for more details.

DAGs, directed acyclic graphs; DREAMS, Determined, Resilient, Empowered, AIDS-free, Mentored and Safe.

**Table 3:** Estimated causal effect of DREAMS on knowledge of HIV status, from regression analysis with adjustment for the ‘propensity to be a DREAMS beneficiary’ score and age, overall and stratified by age group

	% Know status in total study population	Estimated % know status if none benefit from DREAMS	95% CI	Estimated % Know status if all benefit from DREAMS	95% CI	Marginal causal per cent difference (%) (PS adjusted)	95% CI	Prevalence ratio (PS adjusted)	95% CI	OR (PS adjusted)	95% CI	PAF*	95% CI
Nairobi													
Overall	80.67	65.1	(60.9 to 69.5)	92.9	(90.6 to 95.0)	27.77	(22.8 to 32.6)	1.43	(1.33 to 1.53)	6.98	(4.84 to 10.47)	19.3	
15–17 years	75.69	61.1	(53.9 to 67.7)	92.8	(89.9 to 95.2)	31.76	(24.9 to 39.1)	1.52	(1.37 to 1.72)	8.26	(5.46 to 13.83)	19.28	
18–22 years	85.77	70.9	(64.4 to 78.2)	93.5	(89.6 to 97.0)	22.61	(15.0 to 29.7)	1.32	(1.19 to 1.47)	5.93	(3.16 to 13.34)	17.34	
uMkhanyakude													
Overall	44.96	42.30	(39.79 to 44.74)	45.87	(40.75 to 49.97)	3.57	(−1.92 to 8.35)	1.08	(0.96 to 1.20)	1.16	(0.92 to 1.40)	5.90	(2.37 to 9.36)
13–17 years	30.45	26.49	(23.18 to 30.08)	35.43	(32.13 to 38.95)	8.95	(4.81 to 14.41)	1.38	(1.17 to 1.62)	1.52	(1.26 to 2.00)	13.01	(7.41 to 21.74)
18–22 years	61.07	61.15	(57.82 to 64.53)	58.31	(50.24 to 63.90)	−2.84	(−11.11 to 5.67)	0.95	(0.82 to 1.07)	0.89	(61.12 to 1.19)	−0.14	(−2.98 to 2.81)

*PAF estimate as ((% know status in the total study population) – (predicted % who know status if no DREAMs))/% know status in the total study population.

DREAMS, Determined, Resilient, Empowered, AIDS-free, Mentored and Safe; PAF, Population attributable fraction; PS, propensity score.

In uMkhanyakude, there was a smaller increase in knowledge of HIV status by DREAMS invitation, relative to Nairobi, with weak evidence of an overall difference attributable to DREAMS of 3.6% (95% CI −1.9% to 8.4%; table 3). There was evidence that the effect of DREAMS differed by age group, with evidence of effect only among 13–17 years. Specifically, the effect of DREAMS was estimated to increase knowledge of HIV status by 9% (95% CI 4.8% to 14.4%), from 26.5% if no-one was a DREAMS beneficiary to 35.4% if all were a DREAMS beneficiary among 13–17 years, compared with no evidence of effect among 18–22 years (−2.8%, 95% CI −11.1% to 5.7%; table 3, figure 2). The findings were similar using other measures of effect, with differences in odds and prevalence ratios observed among the younger cohort only (table 3).

In both settings, effect estimates generated through alternative approaches to propensity score regression adjustment (ie, stratification and weighting) were similar (online supplemental table S2).

### HIV testing experiences and modalities

In Nairobi, levels of confidence about getting an HIV test and knowledge of where to get tested were very high (90% and 99%, respectively) across age groups and among both beneficiaries and non-beneficiaries of DREAMS (online supplemental table S3a). Testing in the past 12 months was more frequently reported by DREAMS beneficiaries than non-beneficiaries, among both age groups (89% vs 47% among the younger and 90% vs 73% among the older cohort). When asked where they tested most recently, mobile health facilities were more commonly reported among adolescent girls (<18 years) than young women (21% vs 15%), with no differences by DREAMS invitation. Overall, few participants reported home testing (<9%) or self-testing (<1%). DREAMS beneficiaries were more likely than non-beneficiaries to say their last test had been ‘offered’ to them (eg, 52 vs 29% among 15–17 years) or ‘required’ (eg, 29% vs 15% of non-beneficiaries among adolescent girls). Older participants and non-DREAMS beneficiaries more frequently said they ‘had asked for the test’.

Most AGYW in Nairobi (65%) reported that their motivation for testing was to protect or take care of themselves (with higher reporting of this among DREAMS beneficiaries, in both age groups). Testing to protect an unborn/future child was reported mostly by older participants, especially non-DREAMS beneficiaries (13%) vs 5% of beneficiairies. Among those who reported not testing for HIV in the past year (n=125), the most common reason was ‘I don’t think I have HIV’ (68%), an answer more commonly reported by young adolescents (71% vs 63% of older participants), especially adolescents who were not invited into DREAMS (75% vs 46% of DREAMS beneficiaries). Few AGYW (n=11) did not want to know their HIV status.

As in Nairobi, AGYW in uMkhanyakude expressed high levels of confidence about getting an HIV test if they wanted one (93% overall), including visiting a health facility to get tested (online supplemental table S4). Almost all (>95%) felt it is important for people to know their HIV status. Relatively few had tested in a health facility in the past 12 months: 18% of 13–17 years and 41% of 18–22 years. Home-based testing in the past year was higher among the young beneficiaries (27% vs 19% of non-beneficiaries), while there were no differences in recent use of mobile or partner testing by DREAMS invitation. As in Nairobi, few AGYW used HIV self-testing in the past year (<2% overall).

### HTS delivered through DREAMS

Process evaluation data showed that the ways in which HTS were delivered through DREAMS varied considerably in each setting (see box 1 below). In Nairobi, testing services were ‘centralised’ through one implementing agency which offered testing to all AGYW beneficiaries at the time of DREAMS enrolment, regardless of age, circumstance or perceived risk. In contrast, in uMkhanyakude, HTS were delivered by multiple DREAMS implementers and AGYW were linked to testing through community mobilisation events or during SRH or ante-natal care service provision. In both settings, testing was available in community-based settings (eg, ‘safe spaces’ and tents set up for DREAMS in Nairobi and uMkhanyakude, respectively, and some home-based testing) as well as referrals to primary health facilities.

Box 1 **Models of delivering HIV testing through dreams in each setting**

**Nairobi, Kenya**

In the two informal settlement areas of Nairobi, HIV testing services (HTS) were coordinated and delivered by one implementing partner (IP) per area. Both IPs were experienced in HTS provision prior to Determined, Resilient, Empowered, AIDS-free, Mentored and Safe (DREAMS). All adolescent girls and young women (AGYW) who were recruited into DREAMS were eligible for HIV testing and offered testing at the time of an enrolment interview for DREAMS. AGYW received their result at the time of the test and were referred to interventions based on their status and other factors (eg, sexual/pregnancy history, experience of violence). Testing was usually administered in a community site clinic established for DREAMS (in a compound with a coordination office), or in DREAMS ‘safe spaces’, such as community halls, churches and other local venues used for delivery of DREAMS interventions. Caregiver consent was required for those aged <15 years, and girls aged 15+ years could provide their own consent. Family testing services were also available within the homes of DREAMS beneficiaries, to those seeking this service, and walk-in clients (AGYW and males not enrolled in DREAMS) could seek HTS within the site clinics.

**uMkhanyakude, South Africa**

In uMkhanyakude, KwaZulu-Natal, HTS were delivered by multiple DREAMS IPs in the same areas (20 municipal wards). Prior to DREAMS, the IPs were experienced in provision of HTS in other geographical areas, and DREAMS funding enabled expansion into uMkhanyakude. DREAMS IPs used a variety of approaches to offer HTS. For example, some identified AGYW through community mobilisation events and provided information and a referral to testing services. HIV testing was administered to consenting AGYW within communities, for example, in tents, as well as public health care facilities where HIV testing was integrated with sexual reproductive health services and antenatal care. Some IPs provided door-to-door testing, primarily to target male sexual partners of AGYW, but offered HTS to AGYW in the households they visited. IPs that were not contracted to provide HTS issued referrals to AGYW participating in other DREAMS programmes such as school-based or community-based interventions, parenting programmes and other interventions in the DREAMS core package.

## Discussion

### Key findings

In large, representative samples of young women in two diverse settings, we assessed the early impact of the DREAMS Partnership on an important precursor to reducing HIV incidence: knowledge of HIV status. In both settings, we found that exposure to DREAMS (a multisectoral HIV prevention intervention for AGYW) led to an increase in knowledge of HIV status among the younger cohort (those aged <18 years at enrolment). This increase was more substantial in Kenya (32%) compared with South Africa (9%). DREAMS also increased knowledge of HIV status among the older cohort of young women (18–22 years) in Nairobi (by 23%) but not in uMkhanyakude. We have shown in previous research that DREAMS was more effective at recruiting younger adolescents than the older target group in its first year of implementation.^16^ Here, we see how that may translate into greater evidence of impact among the younger adolescent beneficiaries.

The weaker effect of DREAMS on older participants’ knowledge of status could be due to the programme’s poorer reach of this age group in general, as well as the higher availability, accessibility and motivation to use existing HTS among the older group, for example, through existing antenatal care, prevention of mother-to-child transmission programmes, postnatal care and other services already used by young women (especially where pregnancy rates increase rapidly from age 18, as in South Africa).^17^ While DREAMS was still effective at reaching more women >18 years with testing in Nairobi, that is, more than expected without DREAMS, it was not so in uMkhanyakude.

The stronger effect of DREAMS in Nairobi relative to uMkhanyakude is likely to be influenced by the delivery of HIV testing in each context. In Nairobi, HIV testing was offered systematically by one implementing agency to all DREAMS recruits, at the time of enrolment, regardless of age, circumstance or perceived risk and administered in DREAMS-specific sites (community-based clinics and ‘safe space’ venues dedicated to DREAMS beneficiaries). In contrast, HTS through DREAMS were less coordinated and less centralised in uMkhanyakude, and only 42% of all DREAMS beneficiaries knew their HIV status. The increased knowledge of status observed among younger adolescents in uMkhanyakude may have been due to community-mobilisation events (not provided before DREAMS), where they were informed about testing and offered on-site testing in tents, as well as referrals via other DREAMS interventions and increased home-based testing. (Testing in health facilities over the past year did not differ among DREAMS beneficiaries and non-beneficiaries in this setting.) Another contextual difference may be the degree to which DREAMS clients felt able to decline HIV testing. DREAMS’ approach to HTS was pro-active in Nairobi and beneficiaries most often described their latest HIV test as ‘offered’ (45%) or ‘required’ (28%). Among beneficiaries, testing may have been viewed as an entry point to the DREAMS programme and its benefits, and an incentive to HIV testing may have been perceived by potential beneficiaries or their guardians. In uMkhanyakude, where HIV testing through DREAMS was less systematic or proactive, self-initiated testing was more common. Those who were not recently sexually active or perceived themselves to be at low-risk, may not have sought testing even if availability increased.

These findings are consistent with recommendations to boost community-based HIV testing opportunities, as a way to increase uptake among young people.^6^ Offering testing through ‘safe spaces’ for AGYW, mobile units, events, home-based testing, mHealth and peer outreach, provides alternatives to facility-based testing that is not always acceptable, available or affordable to adolescents.^18 19^ Key barriers expressed by adolescents include ‘unfriendly’ services and concerns about confidentiality, and the perceived need to be sick to use primary healthcare facilities.^20^ ‘Enhanced peer outreach’ by HIV-positive peer mobilisers has been shown to improve HIV testing yield (new diagnoses) among key populations, including female sex workers, in DRC and Haiti,^21^ and may be an effective way to encourage adolescents choosing to test for the first time. Integrating HIV counselling and testing with family planning services—demonstrated in the Integra trial in Kenya and Swaziland—can also boost early testing, and simultaneously prevent HIV and unplanned pregnancy.^22 23^ Providing a mix of testing modalities, including HIV self-testing (directly and peer-assisted self-testing)^24 25^ may ensure the choice and confidentiality that adolescents, a heterogeneous group, need in order to be able to access HTS and know their HIV status.

### Strengths and limitations

A strength of the study is that the findings are unlikely to be explained by confounding or selection bias. The representative samples include AGYW who were and were not invited to participate in DREAMS, and we were able to take into account any systematic differences between those groups. We applied a causal inference framework to control for confounding by individual and household characteristics that were anticipated to be determinants of both DREAMS invitation and knowledge of HIV status, and we made comparisons and causal effect estimates that are analogous to an ‘intention to treat’ analysis for a trial design.^11 12^ We estimated several alternative causal effect measures and found a consistent pattern across all. We also compared the main analytical approach (propensity score regression adjustment) with other methods to control for confounding using propensity scores, such as propensity score stratification and weighting, as well as with conventional multivariable logistic regression. Findings were similar across these approaches, giving confidence that the magnitude and direction of the estimated overall causal effect is robust to different analytical approaches, although all relied on the same interview data (to measure outcomes, exposures and confounders) and were thus equally subject to any bias in those data.

Because DREAMS was not a randomised intervention, it was essential to identify key determinants of being a DREAMS beneficiary and we relied on self-reported invitation into DREAMS to distinguish DREAMS beneficiaries and non-beneficiaries. This may have led to misclassification, and possibly have underestimated the percentage of AGYW who were DREAMS beneficiaries, if AGYW did not recall an ‘invitation’ to DREAMS, or they were invited but did not receive any interventions. Our approach also estimated an overall effect of DREAMS regardless of which DREAMS interventions AGYW actually received. Nevertheless, in previous analyses, we found a strong correlation between invitation to DREAMS and uptake of ‘core package’ interventions, with almost all who were invited to DREAMS receiving multiple interventions, and large differences in the number of ‘core package’ interventions received by those invited to DREAMS compared with those who were not invited.^16^

## Conclusion

South Africa and Kenya are experiencing the first and fourth largest HIV epidemics, respectively.^26^ Among the growing number of young people in these and other African countries, new infections will continue as long as knowledge of HIV status—among HIV-positive individuals (‘the First 90%’) and among all individuals regardless of HIV status—remains low. This evaluation of DREAMS’ initial impact demonstrates that adolescent girls can be reached early (before age 18) with HTS and learn their HIV status before first pregnancy and antenatal care—often when many adolescents test for the first time.^6^ Promoting the value of knowing your HIV status and making voluntary testing services available outside of clinical services—where adolescents can be encouraged and not judged for testing—can prepare young people to test more regularly, to protect their own health and plan for their future. In follow-up rounds, we will evaluate the sustained effect of DREAMS on HIV testing, and whether it successfully links young women from testing into treatment and prevention, for an impact on HIV incidence.

**What is already known on this subject**

- Knowledge of one’s HIV status is a critical first step in linking into treatment and prevention services, and stemming onward transmission.
- Testing rates remain comparatively low among adolescents and young people, a rapidly growing demographic group in Africa upon whom HIV epidemic control depends.
- Complex interventions are increasingly recommended for HIV prevention yet few are delivered at scale or evaluated in real-world settings.

**What this study adds**

- We applied a causal inference framework to estimate the causal effect of being a beneficiary of the Determined, Resilient, Empowered, AIDS-free, Mentored and Safe (DREAMS) partnership on knowledge of HIV status.
- DREAMS quickly and substantially increased knowledge of status among adolescent girls (<18 years) in both urban Kenya and rural South Africa.
- The impact of DREAMS was stronger in Kenyan settings, where HIV testing was coordinated by one implementing partner per area, and offered more pro-actively and systematically than in South Africa.
- A large-scale complex intervention implemented in real-world conditions can have direct and early effects on HIV testing uptake among adolescent girls.

## Data Availability

Data are available on reasonable request.
