# Supplementary material for: Early impact of the DREAMS partnership on young women’s knowledge of their HIV status: causal analysis of population-based surveys in Kenya and South Africa
Source: J Epidemiol Community Health. 2021 Sep 13;76(2):158–67. doi: 10.1136/jech-2020-216042 (PMC8762002; doi:10.1136/jech-2020-216042)
Supplement: Supplementary data [file jech-2020-216042supp001.pdf]

File S1 – Statistical Appendix

Justification for Analytical Methods

In the Kenyan setting, a large proportion of adolescent girls and young women (AGYW) received HIV testing, leading to concerns about having a low number of individuals untested compared to the overall sample, especially within strata of age. We thus focussed on regression adjustment for the propensity score as our primary analysis method. Regression adjustment for the propensity score circumvents concern for low events per variable because of the key property of the propensity score: if the covariate set is sufficient to control for confounding, so is the propensity score. Thus, instead of adjusting for a multidimensional set of confounders, we may adjust for a single propensity score.[1] Since there is little concern about over-parameterising the propensity score model, we could theoretically fit a more complex model to estimate the propensity score than could be reasonably used in a standard outcome regression model.

Amongst the sensitivity analyses, to undertake propensity score stratification, we divided the propensity score (PS) into tertiles and estimated the tertile-specific exposure effect, then computed a weighted average of these tertile-specific effects to compare to the results of propensity score adjustment. PS stratification was not used as the principal analysis method given there remains the possibility of residual confounding within strata.

For propensity score weighting, we calculated the inverse probability of being exposed to DREAMS using the estimated propensity score, and weighted each individual according to this probability to create a pseudo-sample that is therefore balanced on the propensity score, and thus on the distribution of confounders. PS weighting was not used as the principal analysis method given concerns about accurate weights for those with low probability of being exposed to DREAMS.

Causal Validity

To interpret the results from the PS regression adjustment as valid causal estimates, five key assumptions should hold: no interference, consistency, positivity, conditional exchangeability, and correct specification of the propensity score model.

In this context, no interference states that whether one individual potentially knows their HIV status is not changed by exposure to DREAMS by another individual. A potential source of interference might be social networks between AGYW. It is possible that some AGYW who received a DREAMS invite further encouraged their peers to receive HIV testing after their own experiences. However, these instances are likely to be limited to a few if any occasions and should not hinder the estimation of a causal effect in this setting.

Consistency refers to an individual's potential outcome under some hypothesised exposure is the same outcome that will be observed under the observed exposure. Rephrased, consistency implies that exposure to DREAMS must be sufficiently consistent and precisely defined such that variation in receipt of the exposure would not result in a different outcome. While there is certainly heterogeneity in receipt of DREAMS programmes and layers, the impact of this heterogeneity on HIV testing is likely to be minimal given the methods of intervention delivery outlined in Box 1. The intervention is well-defined as receipt of an invite or no receipt of an invite, emulating an intention to treat analysis and reflecting real-world variation in uptake of the programme. Future analyses of participation in DREAMS or similar complex interventions should consider the consistency criterion closely.[2]

Positivity states that all individuals must have a non-zero chance of being exposed or unexposed to DREAMS. There is no structural positivity violation in this sample – any of the AGYW could have potentially received DREAMS. There was some targeting of the programme but in terms of where (high-prevalence areas, or 'hot-spots'), the way it was implemented, and the distribution of the propensity scores, this targeting was carried out in such a way that there was never a non-zero probability that an individual in the cohort could receive or not receive DREAMS.

Conditional exchangeability suggests that, conditional on the covariates adjusted for in the analysis, the exposed and unexposed are otherwise exchangeable such that either group could be theoretically be replaced with the other without affecting the outcome. This is loosely equivalent to the requirement of having no residual confounding. Residual confounding can of course, never be ruled out, but the careful construction of a DAG and consideration of major pathways assists in minimising the risk of bias due to a lack of conditional exchangeability.

The propensity score was specified using linear terms, *i.e.*, without fitting any interaction terms. Specifications of the propensity score with interaction terms were explored and made little difference to the distribution of estimated propensity scores, and thus for conceptual simplicity, we chose not to include interaction terms in the propensity score model. While the correct specification of the propensity score is not empirically verifiable, there is no evidence to suggest the specification used would have resulted in a biased estimate compared to other specifications.

References

1. Rosenbaum PRR, D.B. The central role of the propensity score in observational studies for causal effects. *Biometrika*. 1983;70(1): 41-55
2. Rehkopf DH, Glymour MM, Osypuk TL. The Consistency Assumption for Causal Inference in Social Epidemiology: When a Rose is Not a Rose. *Curr Epidemiol Rep*. 2016 Mar;3(1): 63-71
