# Supplementary material for: Early impact of the DREAMS partnership on young women’s knowledge of their HIV status: causal analysis of population-based surveys in Kenya and South Africa
Source: J Epidemiol Community Health. 2021 Sep 13;76(2):158–67. doi: 10.1136/jech-2020-216042 (PMC8762002; doi:10.1136/jech-2020-216042)
Supplement: Supplementary data [file jech-2020-216042supp002.pdf]

Supplementary Information

Table S1(a). Knowledge of HIV Status among young women in Nairobi, Kenya: levels and associations with DREAMS invitation and key confounding variables estimated using conventional logistic regression

	Total N	Knows HIV Status n (%)	Unadjusted OR (95% CI)	Age- and Location-Adjusted OR (95% CI)	Fully Adjusted* OR (95% CI)	p value**
**DREAMS Invitation**						
Not Invited	545	378 (69.4%)	1.00	1.00	1.00	< 0.001
Invited	536	494 (92.2%)	5.20 (3.61, 7.48)	7.32 (4.99, 10.75)	8.69 (5.84, 12.93)	
**Age & Site**						
Age Group						
15-17 Years	547	414 (75.7%)	1.00	1.00	--	--
18-22 Years	534	458 (85.8%)	1.94 (1.42, 2.64)	1.93 (1.41, 2.64)	N/A^	
Age						
15	156	114 (73.1%)	1.20 (1.12, 1.30)	1.20 (1.12, 1.29)	1.06 (0.93, 1.21)	0.41
16	203	152 (74.9%)				
17	188	148 (78.7%)				
18	138	110 (79.7%)				
19	95	83 (87.4%)				
20	102	85 (83.3%)				
21	94	84 (89.4%)				
22	105	96 (91.4%)				
Location						
Korogocho	617	482 (78.1%)	1.00	1.00	1.00	0.30
Viwandani	464	390 (84.1%)	1.48 (1.08, 2.02)	1.45 (1.06, 2.00)	1.22 (0.84, 1.76)	
**Education**						

Birdthistle I, *et al. J Epidemiol Community Health* 2021; 76:158–167. doi: 10.1136/jech-2020-216042

Current School						
Not Currently in School	455	395 (86.8%)	1.00	1.00	1.00	0.80
Currently in School	626	477 (76.2%)	0.49 (0.35, 0.68)	0.68 (0.46, 1.01)	1.07 (0.65, 1.77)	
Highest Education						
Primary Incomplete	125	91 (72.8%)	1.00	1.00	1.00	0.288
Primary Grade 8	217	180 (82.9%)	1.82 (1.07, 3.09)	1.56 (0.90, 2.69)	1.62 (0.87, 3.02)	
Secondary Form 1	154	120 (77.9%)	1.32 (0.76, 2.28)	1.47 (0.84, 2.59)	1.48 (0.77, 2.84)	
Secondary Form 2	187	159 (85.0%)	2.12 (1.21, 3.72)	1.88 (1.06, 3.36)	2.31 (1.19, 4.51)	
Secondary Form 3	150	120 (80.0%)	1.49 (0.85, 2.62)	1.13 (0.63, 2.04)	1.66 (0.82, 3.36)	
Secondary Form 4	198	161 (81.3%)	1.63 (0.96, 2.77)	0.76 (0.42, 1.40)	1.39 (0.67, 2.88)	
Tertiary	50	41 (82.0%)	1.70 (0.75, 3.87)	0.67 (0.27, 1.66)	1.07 (0.37, 3.08)	
**Socio-economic Status**						
Household Assets						
Low Asset Score	360	293 (81.4%)	1.00	1.00	1.00	0.61
Middle Asset Score	361	294 (81.4%)	1.03 (0.71, 1.50)	0.97 (0.67, 1.43)	0.80 (0.51, 1.25)	
High Asset Score	360	285 (79.2%)	0.88 (0.61, 1.27)	0.89 (0.61, 1.29)	0.87 (0.56, 1.34)	
Poverty Perception						
Very poor	139	120 (86.3%)	1.00	1.00	1.00	0.23
Moderately poor	858	690 (80.4%)	0.65 (0.39, 1.09)	0.67 (0.40, 1.13)	0.69 (0.38, 1.25)	
Not poor	84	62 (73.8%)	0.45 (0.22, 0.89)	0.47 (0.23, 0.94)	0.49 (0.22, 1.11)	
Credit Access						
No Access to Credit	550	443 (80.5%)	1.00	1.00	1.00	0.39
Access to Credit	483	391 (81.0%)	1.03 (0.75, 1.40)	1.04 (0.76, 1.43)	1.05 (0.73, 1.50)	
Don't Know	48	38 (79.2%)	0.92 (0.44, 1.90)	0.99 (0.47, 2.08)	1.75 (0.77, 3.98)	
**Sexual/Reproductive History**						
Ever Had Sex						

Birdthistle I, *et al. J Epidemiol Community Health* 2021; 76:158–167. doi: 10.1136/jech-2020-216042

Ever Had Sex	437	401 (91.8%)	1.00	1.00	--	--
Never Had Sex or Undisclosed	644	471 (73.1%)	0.24 (0.17, 0.36)	0.25 (0.16, 0.41)	N/A^^	
Ever Pregnant						
Ever Pregnant	299	282 (94.3%)	1.00	1.00	1.00	< .001
Never Had Sex	644	471 (73.1%)	0.16 (0.10, 0.28)	0.16 (0.09, 0.29)	0.09 (0.04, 0.19)	
Ever had sex, never Pregnant	138	119 (86.2%)	0.38 (0.19, 0.75)	0.37 (0.18, 0.74)	0.32 (0.15, 0.69)	

^ Age was included as a continuous variable

^^ Ever Had Sex was not included in the multivariable models, because of using a "composite" variable combining information on sexual behaviour and pregnancy

* Fully adjusted model is adjusted for the minimal set of confounding variables identified from the DAG (i.e. all variables presented in table; excluding 'Ever Had Sex')

** p value is from likelihood ratio test, from 'fully adjusted' model

Table S1(b). Knowledge of HIV status among young women in uMkhanyakude, South Africa: levels and associations with DREAMS invitation and key confounding variables estimated using conventional logistic regression

		Total N	Knows HIV Status N (%)	Unadjusted OR OR (95%CI)	Age and Location Adjusted OR (95%CI)	Fully Adjusted* OR (95%CI)	p-value
**DREAMS invitation**							
Not invited		1537	711 (46.3%)	1	1	1	0.003
Invited		636	266 (41.8%)	0.84 (0.69, 1.01)	1.27 (1.03, 1.57)	1.40 (1.12, 1.75)	
**Age group**							
13-17		1143	348 (30.4%)	1	1		
18-22		1030	629 (61.1%)	3.58 (3.00, 4.28)	0.81 (0.57, 1.15)		
**Age**							
	13	236	34 (14.4%	1.34 (1.29, 1.39)	1.34 (1.29, 1.39)	1.20 (1.14, 1.27)	<0.001
	14	222	35 (15.8%)				
	15	232	79 (34.1%)				
	16	240	93 (38.8%)				
	17	213	107 (50.2%)				
	18	243	128 (52.7%)				
	19	228	125 (54.8%)				
	20	242	155 (64.1%)				
	21	174	128 (73.6%)				
	22	143	93 (65.0%)				
**Location**							
Rural		1383	627 (45.3%)	1	1	1	0.782
Peri-urban/urban		771	339 (44.0%)	0.95 (0.79, 1.13)	0.97 (0.80, 1.18)	0.97 (0.79, 1.20)	
**Socio-economic Status (Household assets)**							
Low		723	328 (45.4%)	1	1	1	0.939
Middle		743	342 (46.0%)	1.03 (0.84, 1.26)	1.03 (0.83, 1.29)	1.04 (0.82, 1.32)	
High		598	254 (42.5%)	0.89 (0.71, 1.11)	0.94 (0.74, 1.20)	1.01 (0.78, 1.30)	

Birdthistle I, *et al. J Epidemiol Community Health* 2021; 76:158–167. doi: 10.1136/jech-2020-216042

**Currently in school**						
No	538	348 (64.7%)	1	1	1	0.411
Yes	1635	629 (38.5%)	0.34 (0.28, 0.42)	0.94 (0.73, 1.22)	1.19 (0.79, 1.80)	
**Highest level of education achieved**						
Primary	206	53 (25.7%)	1	1	1	0.25
Secondary	1628	716 (44.0%)	2.27 (1.63, 3.14)	1.22 (0.85, 1.75)	1.38 (0.94, 2.04)	
Completed Secondary/Tertiary	337	208 (61.7%)	4.65 (3.18, 6.82)	0.99 (0.63, 1.56)	1.22 (0.72, 2.09)	
**Ever migrated**						
No	1771	741 (41.8%)	1	1		
Yes	402	236 (58.7%)	1.98 (1.59, 2.46)	0.99 (0.77, 1.26)		
**Food insecurity**						
No	1495	612 (40.9%)	1	1		
Yes	678	365 (53.8%)	1.68 (1.40, 2.02)	1.27 (1.04, 1.55)		
**Ever been/currently pregnant**						
No	1570	523 (33.3%)	1	1		
Yes	553	419 (75.8%)	6.26 (5.02, 7.81)	3.58 (2.78, 4.61)		
**Sexual & pregnancy history**						
Ever pregnant	553	419 (75.8%)	1	1	1	<0.001
Never pregnant & sexually active^	248	127 (51.2%)	0.34 (0.24, 0.46)	0.38 (0.27, 0.52)	0.34 (0.24, 0.48)	
Never pregnant & not sexually active	39	20 (51.3%)	0.34 (0.17, 0.65)	0.38 (0.20, 0.75)	0.34 (0.17, 0.67)	
Never had sex	1272	371 (29.2%)	0.13 (0.10, 0.17)	0.23 (0.18, 0.31)	0.21 (0.16, 0.29)	

* Adjusted for the minimal set of confounders from DAG: Age, urban/rural location, SES, education, sexual & pregnancy history (‘Ever been pregnant’ was not included due to the inclusion of the “composite” variable combining information on sexual and pregnancy history).
^ ’Sexually active’ is Yes for those who reported >=1 sexual partner in the previous 12 months.

Birdthistle I, *et al. J Epidemiol Community Health* 2021; 76:158–167. doi: 10.1136/jech-2020-216042

Supplementary S2(a): Knowledge of HIV status: levels and associations with DREAMS invitation and key confounding variables estimated using conventional logistic regression in Nairobi, among 15-17 year olds

	Total N	Knows Status n (%)	Unadjusted OR (95% CI)	Age and Site Adjusted OR (95% CI)	Fully Adjusted* OR (95% CI)	p value**
**DREAMS Invitation**						
Not Invited	225	123 (54.7%)	1.00	1.00	1.00	< 0.001
Invited	322	291 (90.4%)	7.78 (4.94, 12.26)	7.90 (4.99, 12.51)	9.45 (5.82, 15.36)	
**Age & Site**						
Age						
15	156	114 (73.1%)	1.17 (0.91, 1.50)	1.18 (0.92, 1.51)	1.01 (0.73, 1.41)	0.93
16	203	152 (74.9%)				
17	188	148 (78.7%)				
Site						
Korogocho	317	229 (72.2%)	1.00	1.00	1.00	0.91
Viwandani	230	185 (80.4%)	1.58 (1.05, 2.38)	1.59 (1.06, 2.39)	1.03 (0.62, 1.71)	
**Education**						
Current School						
Not Currently in School	88	67 (76.1%)	1.00	1.00	1.00	0.31
Currently in School	459	347 (75.6%)	0.97 (0.57, 1.66)	0.99 (0.57, 1.72)	1.48 (0.70, 3.13)	
Highest Education						
Primary Incomplete	82	52 (63.4%)	1.00	1.00	1.00	0.18
Primary Grade 8	119	87 (73.1%)	1.57 (0.86, 2.87)	1.49 (0.81, 2.75)	1.66 (0.79, 3.46)	
Secondary Form 1	129	98 (76.0%)	1.82 (1.30, 3.34)	1.68 (0.91, 3.10)	1.76 (0.85, 3.64)	
Secondary Form 2	121	101 (83.5%)	2.91 (1.51, 5.62)	2.58 (1.30, 5.12)	3.18 (1.42, 7.15)	
Secondary Form 3	75	61 (81.3%)	2.51 (1.21, 5.24)	2.07 (0.94, 4.55)	2.52 (0.97, 6.54)	

Secondary Form 4	17	12 (70.5%)	1.38 (0.44, 4.31)	1.15 (0.35, 3.77)	3.10 (0.78, 12.31)	
Tertiary	4	3 (75.0%)	1.73 (0.17, 17.39)	1.23 (0.12, 12.86)	3.68 (0.31, 44.13)	
**SES**						
Household Assets						
Low Asset Score	197	157 (79.7%)	1.00	1.00	1.00	0.37
Middle Asset Score	184	131 (71.2%)	0.80 (0.49, 1.32)	0.81 (0.49, 1.34)	0.75 (0.42, 1.36)	
High Asset Score	166	126 (75.9%)	0.63 (0.39, 1.01)	0.65 (0.41, 1.05)	0.67 (0.38, 1.19)	
Poverty Perception						
Very poor	66	54 (81.8%)	1.00	1.00	1.00	0.41
Moderately poor	435	326 (74.9%)	0.66 (0.34, 1.29)	0.67 (0.34, 1.29)	0.59 (0.27, 1.30)	
Not poor	46	34 (73.9%)	0.63 (0.25, 1.56)	0.62 (0.25, 1.55)	0.61 (0.20, 1.84)	
Credit Access						
No Access to Credit	270	199 (73.8%)	1.00	1.00	1.00	0.19
Access to Credit	248	191 (77.0%)	1.20 (0.80, 1.79)	1.21 (0.81, 1.81)	1.10 (0.68, 1.77)	
Don't Know	29	24 (82.8%)	1.71 (0.63, 4.66)	1.61 (0.59, 4.43)	2.77 (0.87, 8.77)	
**Sexual & Pregnancy history**						
Ever Had Sex						
Ever Had Sex	66	60 (90.1%)	1.00	1.00	--	--
Never Had Sex or Undisclosed	481	354 (73.6%)	0.28 (0.12, 0.66)	0.30 (0.12, 0.71)	N/A^^	
Ever Pregnant						
Ever Pregnant	35	30 (85.7%)	1.00	1.00	1.00	< .001
Never Had Sex	481	354 (73.6%)	0.20 (0.02, 1.81)	0.20 (0.02, 1.79)	0.20 (0.02, 1.81)	
Ever had sex, never pregnant	31	30 (96.8%)	0.09 (0.01, 0.69)	0.10 (0.01, 0.72)	0.09 (0.01, 0.69)	

^ Age was included as a continuous covariate

BMJ Publishing Group Limited (BMJ) disclaims all liability and responsibility arising from any reliance placed on this supplemental material which has been supplied by the author(s)

^^ Ever Had Sex was not included in the multivariable models, because of using a “composite” variable combining information on sexual behaviour and pregnancy
* Fully adjusted model is adjusted for the minimal set of confounding variables identified from the DAG (i.e. all variables presented in table; excluding ‘Ever Had Sex’)
** p value is from likelihood ratio test, from ‘fully adjusted’ model

Birdthistle I, *et al. J Epidemiol Community Health* 2021; 76:158–167. doi: 10.1136/jech-2020-216042

BMJ Publishing Group Limited (BMJ) disclaims all liability and responsibility arising from any reliance placed on this supplemental material which has been supplied by the author(s)

Supplementary S2(b): Knowledge of HIV status: levels and associations with DREAMS invitation and key confounding variables estimated using conventional logistic regression in Nairobi, among 18-22 year olds

	Total N	Knows Status n (%)	Unadjusted OR (95% CI)	Age and Site Adjusted OR (95% CI)	Fully Adjusted* OR (95% CI)	p value**
**DREAMS Invitation**						
Not Invited	320	255 (79.7%)	1.00	1.00	1.00	< 0.001
Invited	214	203 (94.9%)	4.70 (2.42, 9.15)	6.10 (3.08, 12.09)	8.87 (4.22, 18.63)	
**Age & Site**						
Age						
18	138	110 (79.7%)	1.25 (1.05, 1.49)	1.24 (1.05, 1.48)	1.08 (0.85, 1.36)	0.53
19	95	83 (87.4%)				
20	102	85 (83.3%)				
21	94	84 (89.4%)				
22	105	96 (91.4%)				
Site						
Korogocho	300	253 (84.3%)	1.00	1.00	1.00	0.35
Viwandani	234	205 (87.6%)	1.31 (0.80, 2.16)	1.26 (0.76, 2.08)	1.32 (0.74, 2.37)	
**Education**						
Current School						
Not Currently in School	367	328 (89.4%)	1.00	1.00	1.00	0.76
Currently in School	167	130 (77.8%)	0.42 (0.26, 0.68)	1.15 (0.96, 1.38)	0.90 (0.45, 1.80)	
Highest Education						
Primary Incomplete	43	39 (90.7%)	1.00	1.00	1.00	0.74
Primary Grade 8	98	93 (94.9%)	1.91 (0.49, 7.48)	1.84 (0.46, 7.30)	1.80 (0.42, 7.74)	
Secondary Form 1	25	22 (88.0%)	0.75 (0.15, 3.67)	0.99 (0.20, 4.93)	0.74 (0.13, 4.34)	
Secondary Form 2	66	58 (87.9%)	0.74 (0.21, 2.64)	0.87 (0.24, 3.14)	1.00 (0.24, 4.14)	

Birdthistle I, *et al. J Epidemiol Community Health* 2021; 76:158–167. doi: 10.1136/jech-2020-216042

Secondary Form 3	75	59 (78.7%)	0.38 (0.12, 1.22)	0.47 (0.14, 1.54)	0.88 (0.22, 3.54)	
Secondary Form 4	181	149 (82.3%)	0.48 (0.16, 1.43)	0.46 (0.15, 1.40)	0.78 (0.22, 2.74)	
Tertiary	46	38 (82.6%)	0.49 (0.14, 1.75)	0.41 (0.11, 1.50)	0.60 (0.13, 2.75)	
**SES**						
Household Assets						
Low Asset Score	164	136 (82.9%)	1.00	1.00	1.00	0.89
Middle Asset Score	194	168 (86.6%)	1.33 (0.75, 2.38)	1.30 (0.72, 2.33)	1.07 (0.55, 2.11)	
High Asset Score	176	154 (87.5%)	1.44 (0.79, 2.64)	1.48 (0.80, 2.73)	1.19 (0.57, 2.49)	
Poverty Perception						
Very poor	73	66 (90.4%)	1.00	1.00	1.00	0.25
Moderately poor	423	364 (86.1%)	0.65 (0.29, 1.49)	0.67 (0.29, 1.54)	0.80 (0.31, 2.05)	
Not poor	38	28 (73.7%)	0.30 (0.10, 0.86)	0.30 (0.10, 0.89)	0.38 (0.11, 1.33)	
Credit Access						
No Access to Credit	280	244 (87.1%)	1.00	1.00	1.00	0.85
Access to Credit	235	200 (85.1%)	0.84 (0.51, 1.39)	0.83 (0.50, 1.38)	0.96 (0.54, 1.71)	
Don't Know	19	14 (73.7%)	0.41 (0.14, 1.22)	0.47 (0.16, 1.41)	1.38 (0.38, 4.96)	
**Sexual & Pregnancy history**						
Ever Had Sex						
Ever Had Sex	371	341 (91.9%)	1.00	1.00	--	--
Never Had Sex or Undisclosed	163	117 (71.8%)	0.22 (0.13, 0.37)	0.23 (0.13, 0.40)	N/A^^	
Ever Pregnant						
Ever Pregnant	268	252 (94.0%)	1.00	1.00	1.00	< .001
Never Had Sex	103	89 (86.4%)	0.40 (0.19, 0.86)	0.39 (0.18, 0.85)	0.40 (0.19, 0.86)	
Ever had sex, never Pregnant	163	117 (71.8%)	0.16 (0.09, 0.30)	0.15 (0.08, 0.31)	0.16 (0.09, 0.30)	

Birdthistle I, *et al. J Epidemiol Community Health* 2021; 76:158–167. doi: 10.1136/jech-2020-216042

^ Age was included as a continuous covariate

^^ Ever Had Sex was not included in the multivariable models, because of using a "composite" variable combining information on sexual behaviour and pregnancy

* Fully adjusted model is adjusted for the minimal set of confounding variables identified from the DAG (i.e. all variables presented in table; excluding 'Ever Had Sex')

** p value is from likelihood ratio test, from 'fully adjusted' model

Supplementary Table S3. Estimated risk differences, risk ratios, and odds ratios from alternative approaches to regression adjustment for the propensity score, considered as sensitivity analyses.

**Analytical approach***	**Estimated % who know status if no DREAMS**	**95% CI**	**Estimated % who know status if DREAMS**	**95% CI**	**Prevalence Difference**	**95% CI**	**Prevalence Ratio**	**95% CI**	**Odds Ratio**	**95% CI**
**Nairobi**										
Unadjusted Logistic Regression	69.40	(65.49, 73.23)	**92.16**	**(89.79, 94.55)**	**22.81**	(18.07, 27.23)	1.33	(1.25, 1.41)	5.20	(3.71, 7.72)
Adjusted Logistic Regression	65.64	(61.59, 69.70)	**92.93**	**(90.42, 94.20)**	**27.29**	(22.65, 31.44)	1.42	(1.33, 1.51)	6.88	(4.80, 9.95)
PS Adjustment^	65.10	(61.10, 68.91)	**92.16**	**(90.43, 94.84)**	**27.77**	(22.84, 32.56)	1.43	(1.33, 1.53)	6.98	(4.84, 10.47)
PS Stratification	65.50	(61.41, 69.58)	**92.82**	**(90.78, 94.94)**	**27.34**	(22.48, 32.19)	1.47	(1.36, 1.63)	7.15	(5.08, 15.65)
PS Weighting	65.04	(60.88, 68.82)	**92.87**	**(90.58, 94.89)**	**27.83**	(22.88, 33.02)	1.43	(1.33, 1.54)	7.00	(4.77, 10.60)
**South Africa**										
Unadjusted Logistic Regression	46.26	(43.84, 48.66)	**41.82**	**(37.84, 45.77)**	**-4.44**	(-8.75, 0.02)	0.90	(0.81, 1.01)	0.84	(0.70, 1.00)
Adjusted Logistic Regression	41.90	(39.43, 44.29)	**47.11**	**(42.49, 51.51)**	**5.21**	(0.25, 10.17)	1.12	(1.00, 1.26)	1.24	(1.00, 1.51)
PS Adjustment^	41.89	(39.53, 44.32)	**46.56**	**(42.22, 50.71)**	**4.67**	(-0.33, 9.30)	1.23	(1.08, 1.41)	1.33	(1.09, 1.67)
PS Stratification	42.17	(39.82, 44.86)	**46.04**	**(41.54, 50.27)**	**3.87**	(-1.12, 8.16)	1.19	(1.03, 1.35)	1.27	(1.01, 1.58)
PS Weighting	41.82	(39.29, 44.31)	**46.34**	**(41.72, 50.84)**	**4.52**	(-0.64, 9.18)	1.11	(0.94, 1.23)	1.20	(0.97, 1.48)

* Each effect measure compares the % who would know their status if nobody received DREAMS to the % who would know their status if everyone received DREAMS (analogous to the interpretation of a trial). All effects obtained are marginal.

^ The effect measure reported in Table 3, repeated here for comparison with alternative approaches.

Birdthistle I, *et al. J Epidemiol Community Health* 2021; 76:158–167. doi: 10.1136/jech-2020-216042

Table S4(a). HIV testing experiences and modalities in Nairobi, overall and by DREAMS invitation stratified by age group

All	Overall		Age Group 15-17				Age Group 18-22			
Blue=Cohort who have not been tested			Invited		Not Invited		Invited		Not Invited	
Red=Cohort who have been tested	N	% (col)	N	% (col)	N	% (col)	N	% (col)	N	% (col)
**Did you use HIV testing services in the past 12 months?**										
Yes	819	75.8	287	89.1	106	47.1	193	90.2	233	72.8
No	125	11.6	19	5.9	42	18.7	13	6.1	51	15.9
Not Ever Used or Not Aware	137	12.7	16	5.0	77	34.2	8	3.7	36	11.3
**Was HIV testing a DREAMS program?**										
Yes	439	40.6	253	78.6	17	7.6	157	73.4	12	3.8
No	258	23.9	34	10.6	60	26.7	36	16.8	128	40.0
Don't know	3	0.3	0	0.0	0	0.0	0	0.0	3	0.9
Didn't use HIV Testing in the past 12 months	381	35.2	35	10.9	148	65.8	21	9.8	177	55.3
**Did you use Partner testing in the past 12 months?**										
Yes	211	19.5	49	15.2	9	4.0	74	34.6	79	24.7
No	51	4.7	5	1.6	4	1.8	10	4.7	32	10.0
Not Ever Used or Not Aware	819	75.8	268	83.2	212	94.2	130	60.7	209	65.3
**Was Partner testing a DREAMS program?**										
Yes	80	7.4	39	12.1	0	0.0	39	18.2	2	0.6
No	98	9.1	10	3.1	6	2.7	34	15.9	48	15.0
Don't know	1	0.1	0	0.0	0	0.0	1	0.5	0	0.0
Didn't use Partner Testing in the past 12 months	902	83.4	273	84.8	219	97.3	140	65.4	270	84.4
**I feel confident that I could visit a health facility or testing center to get an HIV test**										
Not at all true	17	1.6	6	1.9	5	2.2	1	0.5	4	1.3

Hardly true	35	3.2	12	3.7	9	4.0	4	1.9	10	3.1
Moderately true	56	5.2	20	6.2	14	6.2	8	3.7	14	4.4
Exactly true	973	90.0	284	88.2	197	87.6	201	93.9	292	91.3
**Do you know where a person can be tested for HIV?**										
Yes	1,068	98.8	321	99.7	218	96.9	214	100.0	315	98.4
No	13	1.2	1	0.3	7	3.1	0	0.0	5	1.6
**Would you be able to get an HIV test if you wanted one?**										
Yes	1,063	98.3	319	99.1	215	95.6	214	100.0	315	98.4
No	18	1.7	3	0.9	10	4.4	0	0.0	5	1.6
**Is it important for people to know their status?**										
Yes	1,061	98.1	316	98.1	222	98.7	207	96.7	316	98.8
No	20	1.9	6	1.9	3	1.3	7	3.3	4	1.3
**Have you ever been tested for HIV and received the results?**										
Yes	955	88.3	309	96.0	150	66.7	209	97.7	287	89.7
No	126	11.7	13	4.0	75	33.3	5	2.3	33	10.3
**[If never tested] Why have you not tested for HIV?**										
I don't think I have HIV	86	68.3	6	46.2	56	74.7	3	60.0	21	63.6
I don't want to know my HIV status	11	8.7	3	23.1	4	5.3	0	0.0	4	12.1
I don't know where to go for HIV testing	6	4.8	0	0.0	5	6.7	0	0.0	1	3.0
I am worried someone will see me at the clinic and think I am HIV+	0	0.0	0	0.0	0	0.0	0	0.0	0	0.0
I am worried that health workers might reveal my test results to others	0	0.0	0	0.0	0	0.0	0	0.0	0	0.0

Birdthistle I, *et al. J Epidemiol Community Health* 2021; 76:158–167. doi: 10.1136/jech-2020-216042

The clinic where HIV testing is available is far from my house	3	2.4	2	15.4	1	1.3	0	0.0	0	0.0
Other	15	11.9	1	7.7	7	9.3	1	20.0	6	18.2
Don't Know	5	4.0	1	7.7	2	2.7	1	20.0	1	3.0
**[If Ever Tested] Where did your last HIV test take place?**										
At a fixed health facility	677	70.9	214	69.3	89	39.6	166	79.4	208	72.5
At a mobile health facility	188	19.7	65	21.0	49	21.8	26	12.4	48	16.7
At home by provider	81	8.5	27	8.7	10	4.4	16	7.7	28	9.8
At home self testing	7	0.7	3	1.0	1	0.4	0	0.0	3	1.0
At home tested by other person partner relative	2	0.2	0	0.0	1	0.4	1	0.5	0	0.0
**[If Ever Tested] Was the last HIV test offered, required or did you ask for the test?**										
It was offered	430	45.0	159	51.5	66	29.3	94	45.0	110	34.4
It was required	254	26.6	90	29.1	34	15.1	57	27.3	73	22.8
I asked for the test	270	28.3	60	19.4	49	21.8	58	27.8	104	32.5
Don't Know	1	0.1	0	0.0	1	0.4	0	0.0	33	10.3
**[If Ever Tested] What was the main reason for your last HIV test?**										
To protect/take care of yourself	618	64.7	221	71.5	99	44.0	137	65.6	161	50.3
To protect your partner	9	0.9	2	0.6	1	0.4	2	1.0	4	1.3
To protect unborn/future children	69	7.2	6	1.9	9	4.0	11	5.3	43	13.4
To plan for the future	75	7.9	27	8.7	13	5.8	16	7.7	19	5.9
I was sick and worried	21	2.2	1	0.3	5	2.2	3	1.4	12	3.8
I had to confirm my HIV status for PEP	21	2.2	6	1.9	5	2.2	5	2.4	5	1.6
My husband/partner/client wanted to kno	7	0.7	2	0.6	0	0.0	4	1.9	1	0.3
I had unprotected sex	8	0.8	1	0.3	2	0.9	2	1.0	3	0.9
Employment requirement	3	0.3	0	0.0	0	0.0	3	1.4	0	0.0
Other (Specify)	124	13.0	43	13.9	16	7.1	26	12.4	39	12.2

Birdthistle I, *et al. J Epidemiol Community Health* 2021; 76:158–167. doi: 10.1136/jech-2020-216042

**[If Ever Tested] The last (most recent) time you were HIV tested, were you tested for HIV alone or with a sexual partner?**										
Alone	867	90.8	304	98.4	145	64.4	187	89.5	231	72.2
With partner	85	8.9	4	1.3	5	2.2	20	9.6	56	17.5
Tested alone but counselled with partner	3	0.3	1	0.3	0	0.0	2	1.0	0	0.0
**Have you ever tested yourself for HIV using a HIV self-testing kit?**										
Yes	31	2.9	5	1.6	3	1.3	8	3.7	15	4.7
No	760	70.3	223	69.3	156	69.3	150	70.1	230	71.9
Have never heard of HIV self-testing	290	26.8	94	29.2	66	29.3	56	26.2	75	23.4
**If a simple-to-use at home, free HIV test were available to you, how interested would you be in testing yourself for HIV?**										
Very interested	783	72.4	214	66.5	151	67.1	178	83.2	240	75.0
Somewhat interested	97	9.0	32	9.9	26	11.6	13	6.1	26	8.1
Not at all interested	198	18.3	74	23.0	48	21.3	23	10.7	53	16.6
Don't Know	3	0.3	2	0.6	0	0.0	0	0.0	1	0.3
**In your opinion, are people hesitant to take an HIV test because they fear other people's reaction if the test result is positive for HIV?**										
Yes	974	90.1	285	88.5	200	88.9	203	94.9	287	89.7
No	94	8.7	34	10.6	20	8.9	9	4.2	30	9.4
Don't Know/Unsure	13	1.2	3	0.9	5	2.2	2	0.9	3	0.9

Table S4(b). HIV testing experience and modalities in uMkhanyakude, overall and by DREAMS invitation stratified by age group.

	Overall		13-17				18-22			
			Invited		Not invited		Invited		Not invited	
	N	% (col)	n	% (col)	n	% (col)	n	% (col)	n	% (col)
**Did you use facility-based HIV testing services in past 12 months**										
Yes	623	28.6	84	18.1	118	17.3	80	46.0	341	39.7
No	208	9.5	28	6.0	37	5.4	29	16.7	114	13.3
Not ever used or not aware	1349	61.9	351	75.8	529	77.3	65	37.4	404	47.0
**Was the facility-based HIV testing a DREAMS program?**										
Yes	126	5.8	39	8.4	30	4.4	20	11.5	37	4.3
No	492	22.6	45	9.7	86	12.6	60	34.5	301	35.0
Don't know	5	0.2	0	0	2	0.3	0	0	3	0.3
Didn't use HIV testing in past 12 mo	1557	71.4	379	81.9	566	82.7	94	54	518	60.3
**Did you use Partner testing in past 12 months?**										
Yes	129	5.9	9	2.0	12	1.8	19	10.9	89	10.4
No	40	1.8	0	0	4	0.6	7	4	29	3.4
Not used or not aware	2003	92.2	451	98.0	666	97.7	149	85.1	737	86.2
**Was Partner testing a DREAMS program?**										
Yes	24	1.1	6	1.3	7	1	1	0.6	10	1.2
No	103	4.7	3	0.7	5	0.7	18	10.3	77	9
Don't know	2	0.1	0	0	0	0	0	0	2	0.2
Didn't used partner testing in past 12 months	2043	94.1	451	98	670	98.2	156	89.1	766	89.6
**Did you use home testing in past 12 months?**										
Yes	766	35.3	122	26.5	128	18.9	82	47.4	434	50.6
No	165	7.6	11	2.4	17	2.5	18	10.4	119	13.9
Not ever used or not aware	1239	57.1	327	71.1	534	78.6	73	42.2	305	35.5

Birdthistle I, *et al. J Epidemiol Community Health* 2021; 76:158–167. doi: 10.1136/jech-2020-216042

**Was Home testing a DREAMS program?**										
Yes	140	6.5	32	7.0	31	4.6	21	12.1	56	6.5
No	620	28.6	88	19.1	98	14.4	61	35.3	373	43.5
Don't know	7	0.3	2	0.4	0	0	0	0	5	0.6
Didn't use home testing in past 12 months	1403	64.7	338	73.5	550	81	91	52.6	424	49.4
**Did you mobile testing in past 12 months?**										
Yes	132	6.1	12	2.6	22	3.2	11	6.3	87	10.2
No	173	8	19	4.1	19	2.8	37	21	98	11.5
Not ever used or Not aware	1868	86	431	93.3	639	94	128	72.7	670	78.4
**Was Mobile testing a DREAMS program?**										
Yes	25	1.2	3	0.6	3	0.4	5	2.8	14	1.6
No	99	4.6	8	1.7	16	2.4	6	3.4	69	8.1
Don't know	9	0.4	1	0.2	4	0.6	0	0	4	0.5
Didn't use mobile testing in past 12mo	2040	93.9	450	97.4	657	96.6	165	93.8	768	89.8
**Did you use Self testing in past 12 months?**										
Yes	34	1.6	4	0.9	6	0.9	3	1.7	21	2.5
No	20	0.9	1	0.2	3	0.4	2	1.1	14	1.6
Not ever used or not aware	2119	97.5	455	98.9	672	98.7	171	97.2	821	95.9
**Was Self testing a DREAMS program?**										
Yes	7	0.3	4	0.9	1	0.1	1	0.6	1	0.1
No	26	1.2	0	0	4	0.6	2	1.1	20	2.3
Don't know	1	0	0	0	1	0.1	0	0	0	0
Didn't use self-testing in past 12mo	2139	98.4	456	99.1	675	99.1	173	98.3	835	97.5
**I feel confident that I can visit health facility to get an HIV test**										
Definitely not true	45	2.1	15	3.2	9	1.3	3	1.7	18	2.1
Slightly true	114	5.2	15	3.2	32	4.7	12	6.8	55	6.4
Probably true	298	13.7	41	8.9	63	9.2	8	4.5	186	21.7

Definitely true	1725	79.1	392	84.7	581	84.8	153	86.9	599	69.8
**I feel confident that I would be able to go to a health clinic for any reason, if I wished to**										
Definitely not true	30	1.4	6	1.3	11	1.6	0	0	13	1.5
Slightly true	116	5.3	20	4.3	25	3.6	8	4.5	63	7.3
Probably true	278	12.7	45	9.7	65	9.5	12	6.8	156	18.2
Definitely true	1759	80.6	392	84.7	584	85.3	156	88.6	627	73
**Would you be able to get HIV test if you wanted one?**										
Yes	2030	93	430	92.9	630	92.0	168	95.5	802	93.4
No	142	6.5	33	7.1	52	7.6	6	3.4	51	5.9
Prefer not to answer	11	0.5	0	0	3	0.4	2	1.1	6	0.7
**Is it important for people to know their status?**										
Yes	2080	95.3	448	96.8	640	93.4	171	97.2	821	95.6
No	93	4.3	13	2.8	42	6.1	4	2.3	34	4
Prefer not to answer	10	0.5	2	0.4	3	0.4	1	0.6	4	0.5
**Have you ever been tested for HIV and received the results**										
Yes	1337	61.5	223	48.5	253	37.0	134	76.1	727	85.1
No	828	38.1	236	51.3	428	62.7	41	23.3	123	14.4
Prefer not to answer	8	0.4	1	0.2	2	0.3	1	0.6	4	0.5
**Where did your last HIV test take place?**										
Health facility/clinic	935	69.9	108	48.2	158	62.5	95	70.9	574	79
Mobile clinic	108	8.1	33	14.7	23	9.1	9	6.7	43	5.9
Home	254	19	68	30.4	68	26.9	26	19.4	92	12.7
Other, specify	41	3.1	15	6.7	4	1.6	4	3	18	2.5
**Was the last HIV test offered, required or did you ask for the test?**										
Offered	367	27.4	119	53.1	85	33.6	31	23.1	132	18.2
Required	376	28.1	34	15.2	55	21.7	37	27.6	250	34.4

Asked for it	595	44.5	71	31.7	113	44.7	66	49.3	345	47.5
**What was the main reason for your last HIV test?**										
To protect myself	707	52.8	137	60.9	159	62.8	50	37.3	361	49.7
To protect partner	7	0.5	0	0	2	0.8	1	0.7	4	0.6
To protect unborn child	167	12.5	7	3.1	15	5.9	14	10.4	131	18.0
To prepare for the future	212	15.8	35	15.6	39	15.4	17	12.7	121	16.7
Other, specify	245	18.3	46	20.4	38	15.0	52	38.8	109	15.0
**Did you receive any counselling prior to your last HIV test?**										
Yes	1142	85.4	193	86.2	221	87.4	122	91.0	606	83.4
No	191	14.3	29	12.9	32	12.6	11	8.2	119	16.4
Prefer not to answer	5	0.4	2	0.9	0	0	1	0.7	2	0.3
**Have you ever had a positive HIV test result?**										
Yes	129	9.3	10	4.2	15	5.7	13	8.9	91	12.4
No	1241	89.8	227	94.6	247	93.6	132	90.4	635	86.7
Prefer not to answer	12	0.9	3	1.3	2	0.8	1	0.7	6	0.8

Birdthistle I, *et al. J Epidemiol Community Health* 2021; 76:158–167. doi: 10.1136/jech-2020-216042
